# Concepts, utilization, and perspectives on the Dutch Nationwide Trauma registry: a position paper

**DOI:** 10.1007/s00068-022-02206-4

**Published:** 2023-01-09

**Authors:** R. J. Hoepelman, M. L. S. Driessen, M. A. C. de Jongh, R. M. Houwert, I. Marzi, F. Lecky, R. Lefering, B. J. M. van de Wall, F. J. P. Beeres, M. G. W. Dijkgraaf, R. H. H. Groenwold, L. P. H. Leenen

**Affiliations:** 1 Department of Surgery, University Medical Center Utrecht, PO Box 85500, 3508 GA Utrecht, The Netherlands; 2 Dutch Network for Emergency Care (LNAZ), Utrecht, The Netherlands; 3 Brabant Trauma Registry, Network Emergency Care Brabant, Tilburg, The Netherlands; 4 Department of Trauma, Hand and Reconstructive Surgery, University Hospital Frankfurt, Goethe-University, Frankfurt, Germany; 5 The Trauma Audit and Research Network, The University of Manchester, Salford Royal-Northern Care Alliance NHS Foundation Trust, Salford, UK; 6 Centre for Urgent and Emergency Care Research, Health Services Research Section, School of Health and Related Research, University of Sheffield, Sheffield, UK; 7 Faculty of Health, IFOM-Institute for Research in Operative Medicine, University Witten/Herdecke, Cologne, Germany; 8 Department of Orthopaedic and Trauma Surgery, Lucerne Cantonal Hospital, Lucerne, Switzerland; 9 Department of Epidemiology and Data Science, Amsterdam UMC, Amsterdam, The Netherlands; 10 Department of Methodology, Amsterdam Public Health, Amsterdam, The Netherlands; 11 Department of Clinical Epidemiology, Leiden University Medical Center, Leiden, The Netherlands; 12 Department of Biomedical Data Sciences, Leiden University Medical Center, Leiden, the Netherlands

**Keywords:** Trauma registry, Trauma systems, DNTR, Trauma surgery

## Abstract

Over the last decades, the Dutch trauma care have seen major improvements. To assess the performance of the Dutch trauma system, in 2007, the Dutch Nationwide Trauma Registry (DNTR) was established, which developed into rich source of information for quality assessment, quality improvement of the trauma system, and for research purposes. The DNTR is one of the most comprehensive trauma registries in the world as it includes 100% of all trauma patients admitted to the hospital through the emergency department. This inclusive trauma registry has shown its benefit over less inclusive systems; however, it comes with a high workload for high-quality data collection and thus more expenses. The comprehensive prospectively collected data in the DNTR allows multiple types of studies to be performed. Recent changes in legislation allow the DNTR to include the citizen service numbers, which enables new possibilities and eases patient follow-up. However, in order to maximally exploit the possibilities of the DNTR, further development is required, for example, regarding data quality improvement and routine incorporation of health-related quality of life questionnaires. This would improve the quality assessment and scientific output from the DNTR. Finally, the DNTR and all other (European) trauma registries should strive to ensure that the trauma registries are eligible for comparisons between countries and healthcare systems, with the goal to improve trauma patient care worldwide.

**Supplementary Information:**

The online version contains supplementary material available at 10.1007/s00068-022-02206-4.

## Introduction

The Dutch trauma system has been subject to major improvements in the past two decades, and as a result, significant reductions in the overall mortality and morbidity of trauma patients were recorded. This is mostly attributable to the improvements to the organizational structure of the trauma system and to the acute trauma and intensive care management in general [1, 2].

The ultimate way to determine the performance of the entire trauma system is through adequate and complete patient-level data from trauma registries [3]. Therefore, the Dutch Nationwide Trauma Registry (DNTR) was established, to measure, evaluate, and further improve care for Dutch trauma patients [4]. Furthermore, the comprehensive and prospectively gathered data gives the opportunity to assess a variety of research questions, such as epidemiological changes during a pandemic, evaluation of the provided trauma care, or asses the effect of treatment of a specific injury pattern [5–8]. The purpose of this position paper is to give an overview of the Dutch trauma system and the DNTR in particular. Primarily to help others, interpret studies based on trauma registries, provide them with guidance to overcome obstacles, and avoid pitfalls in their own registries or research projects.

### History of the Dutch trauma system and the DNTR

Before 1999, the Dutch trauma system was nonintegrated and unorganized. Research described in a PhD thesis in 1987 revealed that—at that time—trauma patients were generally transported to the closest hospital, rather than the most appropriate hospital [9]. This thesis and a mid-city airplane crash in the Netherlands in 1992 were the main catalyst that started the development of regionalized trauma care, designated major trauma centers, and the use of field triage protocols. The Dutch trauma system was reformed in 1999, following the American College of Surgeons Committee on Trauma (ASCOT) guidelines entitled “Optimal Resources for Care of the Seriously injured.” [10]. This structural and logistical optimization has had a major impact on trauma care in the Netherlands, as it did in other countries that implemented the inclusive trauma systems [11, 12]. Along with these organizational changes, the changes in treatment approach of severely injured patients was of significant contributory importance [1, 3]. This led to an absolute region-wide reduction in crude mortality of 50% and an absolute reduction in pre-hospital deaths due to exsanguination of 40% [1].

In 2007, the DNTR was established to determine the performance of the renewed Dutch trauma system. The primary goal was quality registration to improve care for trauma patients in the Netherlands. The data collected within the DNTR provide a comprehensive source of information for quality assessment, quality improvement, policy makers, and research purposes.

### The current Dutch trauma system

There are eleven level-1 Regional Trauma Centers (RTC) which form geographically defined inclusive trauma regions. In each region, these centers fulfill a coordinating role that encompasses multiple level-II and III trauma centers. To assist patients’ triage to the appropriate level of care, ambulance services are assisted by a trauma field triage decision scheme based on vital signs, injury type, and mechanism of injury [13]. To further improve prehospital triage, the Trauma Triage App has been developed, which is currently in trial [14]. The level-I trauma centers are fully equipped to deliver the highest level of emergency and surgical care for the most severely injured with 24/7 coverage of all specialties including thoracic and neurosurgery. Within the regional trauma systems, all trauma-receiving hospitals have a direct linkage to a RTC, to facilitate expeditious transfer of injured patients within the network, to the hospital with the medical expertise and instrumental capacity that matches their alleged resource needs. Additionally, four of these RTCs are equipped with 24/7 Helicopter Emergency Medical Service (HEMS) and a Mobile Medical Team (MMT) which are able to dispatch by helicopter or ground vehicle. Lower-level trauma centers (i.e., level-II and level-III), on the other hand, were established to provide optimal care for moderately and mildly injured patients in a cost-effective manner. The current composition of trauma patients admitted to level-I and level-II and III trauma centers is described in Table 1.

**Table 1:** Characteristics of acutely admitted trauma patients in the Netherlands for the year 2021 in Regional Trauma Centers (RTC) and Non-RTCs

	All patients *N* = 72,371	RTC *N* = 17,225	Non-RTC *N* = 55,146
Mean age (SD)	66 (31–81)	56 (24–75)	69 (36–83)
Male sex	35,782 (49.4%)	9958 (57.8%)	25,824 (46.8%)
Median LOS (days, IQR)	4 (2–7)	3 (2–8)	4 (2–7)
Blunt trauma	65,281 (90.2%)	16,176 (93.9%)	49,105 (97.2%)
Median ISS (IQR)	6 (4–9)	9 (4 -12)	6 (4–9)
AIS severity score and region of injury			
Head ≥ 3	5279 (7.3%)	2730 (3.8%)	2549 (3.5%)
Face ≥ 3	214 (0.3%)	158 (0.2%)	56 (0.1%)
Neck ≥ 3	71 (0.1%)	56 (0.1%)	15 (0.0%)
Upper extremities ≥ 3	313 (0.45)	163 9 (0.2%)	2255 (3.1%)
Thorax ≥ 3	4310 (6.0%)	2055 (2.8%)	306 (0.4%)
Spine ≥ 3	1229 (1.7%)	711 (1.0%)	518 (0.7%)
Abdomen ≥ 3	768 (1.1%)	462 (0.6%)	150 (0.2%)
Lower extremities ≥ 3	23,135 (32.0%)	3934 (5.4%)	19,201 (26.5%)
External ≥ 3	380 (0.5%)	221 (0.3%)	159 (0.2%)
ICU admission	4797 (6.6%)	2940 (17.1%)	1857 (3.4%)
Median ICU LOS (IQR), (days)	2 (1–3)	2 (2–3)	2 (1–3)
In-hospital mortality	1948 (2.7%)	835 (4.8%)	1113 (2.0%)
30D mortality	3080 (4.2%)	980 (5.6%)	2100 (3.8%)

LOS, length of stay; ICU, Intensive Care Unit; ISS, Injury Severity Score; AIS, Abbreviated Injury Score; 30D mortality, patients that within thirty days after injury

In contrast to most other countries, Dutch trauma surgeons are general surgeons with a specialization in trauma orthopedics. As a result, Dutch trauma surgeons treat both visceral and extremity injuries, and this patient mix is therefore represented in the DNTR [3].

### Who is in the DNTR?

The DNTR includes all injured patients who are directly admitted to the hospital through the Emergency Department (ED), transferred to another hospital, deceased during ER treatment, or within 48 h after trauma. Patients declared dead before hospital arrival or without vital signs upon arrival at the ED are not included.

**Fig. 1:**
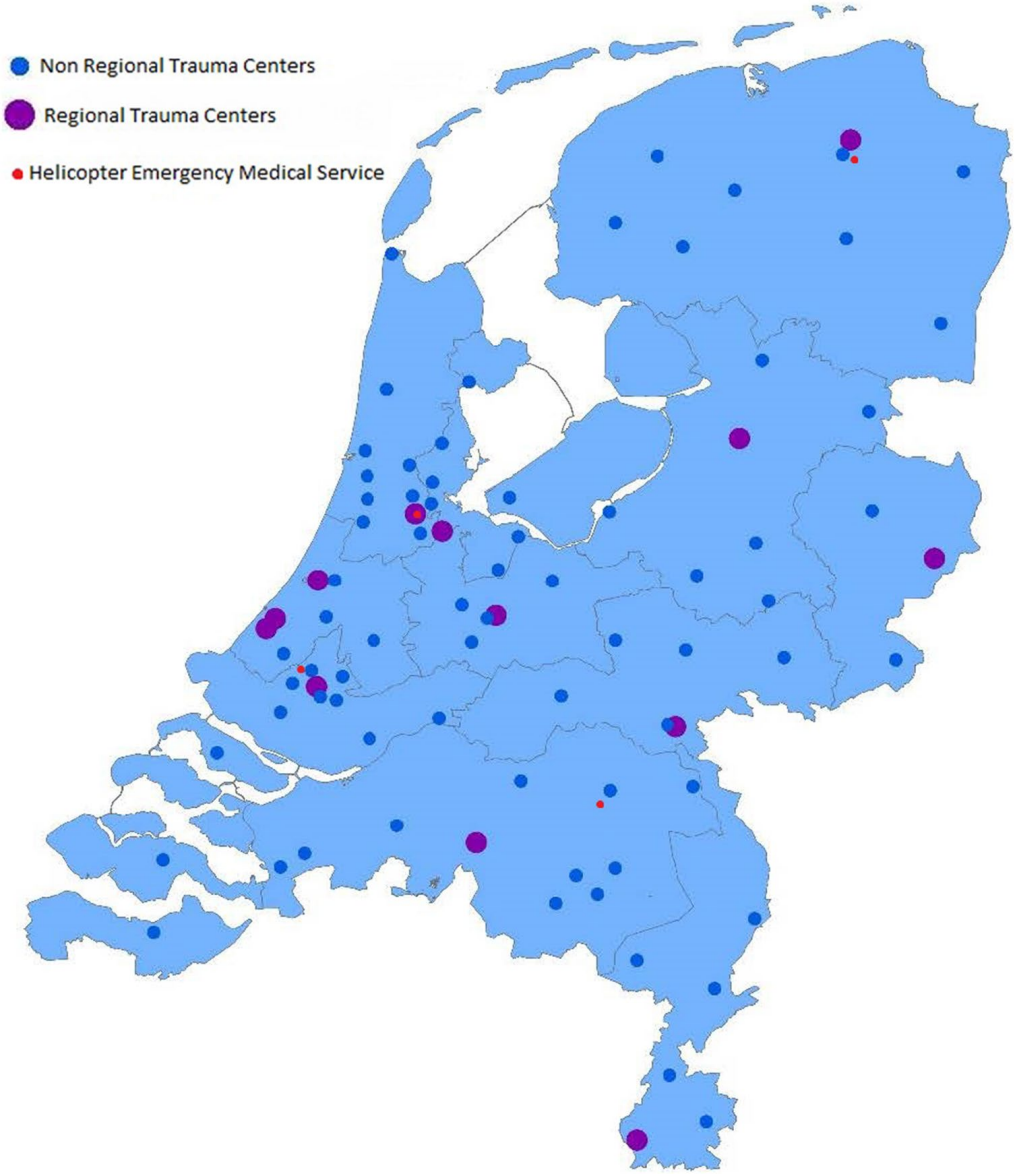
Distribution of Regional Trauma Centers (RTC), Helicopter Emergency Medical services (HEMS) and non-RTCs with emergency departments in the Netherlands

**Fig. 2:**
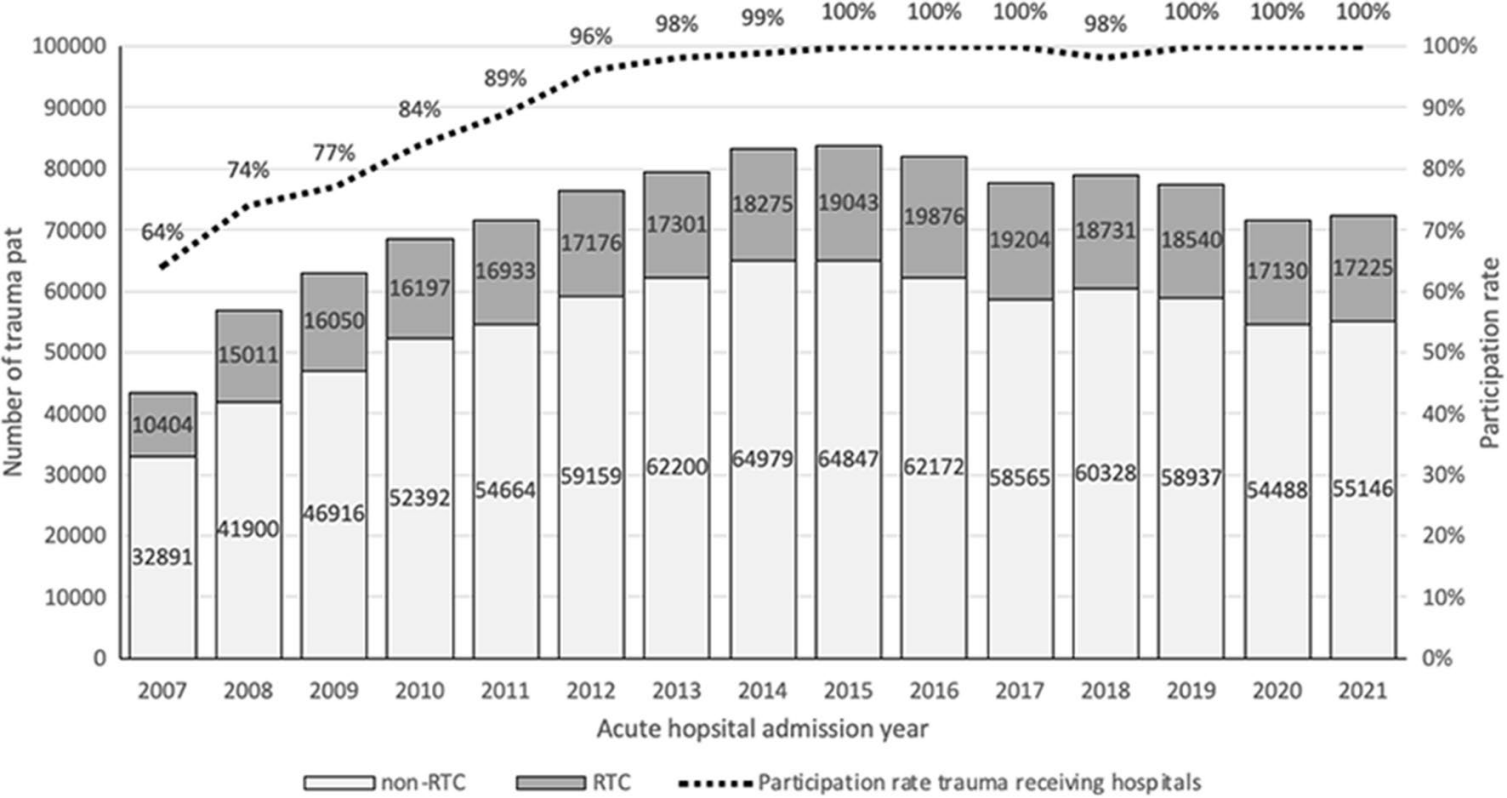
Number of acute trauma admission registered by the Regional Trauma Centers (RTC), the non-Regional Trauma Centers and the hospital participation rate in the Dutch National trauma Registry, 2007–2021. *Due to the closure of two Emergency Departments the participation rate fell to 98%

In 2022, there were 86 trauma receiving hospital in the Netherlands. Thirteen were devoted level-I trauma centers, of which eleven acted as an RTC (Fig 1). Between 2007 and 2021, a total of 1.087.046 trauma cases were registered in the DNTR. In 2007, 64% of all Dutch hospitals with an ED participated. Since 2015, the participation rate increased to 100% as shown in Fig. 2.

### Which information is registered in the DNTR?

In 2007, the Utstein template for Uniform Reporting of Data following Major Trauma was established, which formed the basis for the DNTR. The Utstein template suggested that trauma registries should differentiate between data variables that absolutely need to be collected (core data) and the type of additional data that may be desirable (optional data) [15]. The core data of the Utstein template were divided into three groups (‘Predictive Model,’ ‘System Characteristic Descriptors’ and ‘Process Mapping Variables’) based on the role of the data variable in a registry. The complete set of the currently recorded variables in the DNTR is available in Supplementary Table 1–3. Outcomes measures collected in the DNTR include hospital length of stay, intensive care unit length of stay, duration of mechanical ventilation, need for surgery, in-hospital mortality and 30-day mortality. (Supplementary Table 2–3).

### Data collection and data quality

Data collection is done by trained personnel or trained medical professionals that work according to a strict protocol. The DNTR is embedded in a web-based relational database (SQL). A trusted third party secures privacy sensitive information and encrypts personal data. Data can be entered through an online data entry application with plausibility checks or by import of an electronic file. The DNTR organizes data entry training sessions to improve the quality of data and attain a high level of knowledge, for instance, e-learnings are made available for learning AIS coding. The system (although basally) regularly checks data quality and outliers; furthermore, during every annual report, results are thoroughly analyzed, and data are checked and validated in case of unexpected outcomes.

Missing values are an indisputably problem of many trauma registries. Yet, before jumping to conclusions or an imputation method, a researcher needs to be aware on how the variable was recorded and which population it concerns. The percentages per variable in the DNTR are listed in Supplementary Table 1–3. Some variables might seem to have a concerning high number of missing values, yet this specific variable might be clinically irrelevant or only relevant for a small subpopulation.

Methodological support should be consulted as there are various methods (e.g., multiple imputation) to deal with missing values and depending on the type of study one method might be more desirably than another. Both multiple imputation and a complete case analysis are viable methods under certain conditions. An alternative imputation using healthy values (e.g., if the GCS is missing, the highest possible score of 15 is recorded) provides a fast and relatively easy option. Although one should keep in mind that depending on the imputed variables, this results in underestimation of trauma, physiological disturbance, frailty, or mortality, but in some situations such a ‘best-case scenario’ might provide valuable insights.

### Obstacles and possibilities

Although the DNTR has already demonstrated its added value for multiple purposes, further development is required [4, 5, 13]. The DNTR aims to record data from the entire acute care chain, starting at the site of injury until hospital discharge or assessment of the 30-day mortality. Prehospital data are subject to improvement, for which collaboration with prehospital personal [e.g. Emergency Medical Technicians (EMT)] should be refined. Furthermore, personal patient data are required in order to follow a patient through the chain (pre-hospital, (inter)hospital, and after discharge). To ensure the quality of the trauma registry, it was decided that individual patients’ consent was not feasible for several reasons; it is time-consuming for the patient and physician, it would greatly increase administrative tasks, and it could potentially result in selection bias. In particular, this selection bias should be avoided if the register is used to asses quality of the entire trauma system. Therefore, it was decided that no individual consent was needed for patients to be registered in the DNTR; patients do, however, have the possibility to ‘opt out.’ [16]. Naturally, the DNTR complies with the general data protection regulation (GDPR). Previously, the DNTR data were pseudonymized at a regional level and uploaded in the DNTR database, making it practically impossible to link data from different health-care providers to a specific patient. Gladly, new Dutch legislations allow the DNTR to include the required citizen service numbers. Only the DNTR has access to the non-pseudonymized data, and privacy is warranted. Researches requesting data from the DNTR for research purposes will not receive reducible data. This recent development offers new opportunities to present individually collected data on an aggregated level, assess the weaknesses, strengths and make substantiated decisions on the future direction of the acute care chain. Furthermore, it might enable us to link citizen service numbers to different data sources, hereby enlarging the available data and further increase the versatility of the data.

### Comparison to other trauma registries

The Dutch registry differs from other European national registries by capturing all acute trauma-related hospital admissions regardless of their age, injury type or severity, resource use, or length of stay. Inclusion of all acute trauma admissions has value over more strict inclusion criteria, which results in a very restricted view on the magnitude and impact of injury [4]. Focusing solely on injury severity would result in exclusion of a large percentage of especially elderly and infants. Furthermore, we found that selection of only severely injured and intensive care admitted patients encompasses only 5% of all admitted trauma patients in the Netherlands and would leave out almost 70% of fatalities [4]. Finally, using these criteria would result in a gross underestimation of medical resource utilization in the Netherlands. Capturing all acute trauma admissions is a prerequisite to effectively evaluate our trauma systems’ performance, and it enables policy-makers to make weighted decisions on trauma prevention and control and allocation of workforce and resources [4].

It must be said that registering all acute trauma admissions results in a higher workload and thus more expenses. The demand on data managers for the DNTR is already high, while compared to, for instance, the Trauma register of the German Trauma society (TR-DGU), the number of participating hospitals is rather small. It begs the question whether an all-inclusive system as ours is feasible or at the least cost-effective for larger trauma registries.

### Examples of recent research activities using DNTR data

All data in the DNTR are gathered prospectively, however, most conducted studies are observational retrospective cohort studies. Until recently, one of the biggest flaws was the inability to include follow-up parameters (e.g., PROMS) in research projects. Most RTCs have started performing routine EQ5D-based health status follow-up for their trauma admissions, but with the new Dutch legislation, the completeness of these follow-up questionnaires is expected to improve even more. These developments will be a great tool to evaluate the quality of trauma and give rise to new possibilities for research to be conducted on data from the DNTR.

Prediction models were already feasible from the DNTR. The scope of possibilities that have now opened up need to be explored in the near future. Studies will always be limited to the included patients, selection criteria, and variables available. Furthermore, 100% of the key variables should be available; otherwise, records should be excluded. Several different methods for data handling were discussed previously.

Recently, the funnel plot methodology was introduced in the Netherlands to monitor and evaluate trauma care on a hospital level [17]. Using a graphical presentation of the standardized mortality ratio (SMR) (i.e., the ratio of observed deaths to the expected number of deaths or the observed mortality rate to the expected mortality rate), the hospital specific quality of trauma care can be monitored [17, 18].

The prediction of survival probabilities for individual trauma patients is essential for trauma system evaluation. Various models have been developed since the introduction of the Trauma Injury and Severity Score (TRISS) [19]. In contrast to other models, the Dutch model (mTRISS-NL) was developed to accurately predict the survival of all acutely admitted trauma patients using widely available core variables. The mTRISS-NL model includes the variables: sex, American Society of Anaesthesiologists physical status and nonlinear transformations of age, systolic blood pressure, Injury Severity Score and the Glasgow coma scale-derived best motor response [20].

All research proposals on the DNTR need to be approved by the Dutch scientific research board. DNTR never provides exports of data and conducts the research analysis themselves. A well-planned study script describing the variables used in the analysis needs to be provided by the researchers, preferably tested in their regional data. It is recommended to consult an epidemiologist before admitting a research proposal and to assist during the (methodological) process of the study.

### DNTR organization

For the DNTR a board, a scientific advisory committee, a data manager platform, and a program manager have been appointed. Furthermore, the Dutch Trauma Centre Council, composed of leading trauma surgeons from the 11 RTCs, provides their advice for future development and research questions of interest to be performed on the trauma registry. The RTCs receive annual governmental funding to cover expenditures of DNTR infrastructure and wages, providing continuity in sustaining and developing the registry system. One data manager per trauma center, responsible for the coordination of the regional trauma registry, participates in the national data manager platform. Quarterly, the platform discusses cases and definitions of data items to ensure consistency across the regional trauma registries. Furthermore, an online reporting tool is available for the participants including hospital, regional and national benchmark data. Annually national and regional reports are published and handed out at a national conference about the trauma registry results.

### Future prospects/perspectives

#### Data quality enhancements

Currently, the percentages of missing values, as shown in Supplementary Tables 1–3, indicate that there is room for improvement on completeness and consistency of the trauma registry in the Netherlands and registries in general [21, 22]. To address these issues in the future, the DNTR and other trauma registries need to transition from labor-intensive and inefficient data entry and strive for more automated techniques based on electronic health record data and other existing platforms. This will reduce the number of missing values, lower the workload and expand datasets.

Enforcing the completeness and reliability of pre-hospital DNTR data can be also be realized by strengthening the collaboration with regional Emergency Medical Services (EMSs), Mobile Medical Teams and rehabilitation clinics. This collective approach would greatly improve our understanding of the decisions made in a pre- and post-hospital setting, and their effect on a patients’ outcome. These data facilitate improvements in triage protocols and offer the opportunity to initiate EMS feedback loops on whether the patient was transferred to the most appropriate hospital [13]. Most importantly, these developments will lead to better functioning trauma systems and less secondary transport. This will eventually lead to improved patient outcomes and more cost-effective trauma care [20].

Another field of interest is to include a new set of biochemical markers because trauma diagnostics, and especially prognostics, are becoming less reliant on clinical information, and several hematological and biochemical markers have been associated with injuries and worsening outcomes after trauma [23, 24]. For example, prehospital lactate measurement can indicate the need for immediate interventions for correcting haemostasis [25]. Lastly, data obtained from laboratory results are less influenced by subjective assessments of vital signs, which are less reliable in case of intubation, sedation, or intoxication [26]. Structural testing of predefined laboratory diagnostics sets for certain different trauma patient subpopulations could increase the understanding of the physiological changes that occur after trauma, which in turn could serve as the cornerstone in optimizing patient resuscitation and consequently their outcome.

#### Improving evaluation of care

Implementation of a non-fatal outcome measures could be another improvement to the trauma registry. The primary performance indicator for trauma systems has long been mortality; however, 97% of the patients registered in the DNTR have a non-fatal outcome. Studies have shown that trauma patients are significantly impaired on mobility, self-care, and pain up to one year after trauma [21, 27, 28]. Moreover, two years after injury only 23% of the severely injured patients returned to their pre-injury level of function and 70% resumed prior employment status [29, 30]. As a result, the socioeconomic impact of trauma due to health-care dependence and a partial or complete inability to work is extremely high [31]. Therefore, identifying prognostic factors associated with return to work or decreased HRQOL after injury is crucial for quality of care improvements.

Furthermore, since functional outcome assessment is becoming increasingly important in the evaluation of trauma care, the DNTR has started implementation of PROMS into the national registry, measuring HRQOL using the EuroQOL5-Dimensions (EQ5D-5L) and Euro QOL visual analog scale, and a question on cognitive functioning. Nowadays, most of the RTC’s perform routine follow-up of their trauma patients, but not all data are transferred to the DNTR. The standardized addition of PROMS to all DNTR patients would generate invaluable data for the evaluation and optimization of future trauma care. Routine collection of PROMS has proven feasible within European health systems. Response rate in the Netherlands was 75% when paper and telephone questionnaires were performed, without incentive [21]. Besides generating insight in the morbidity of the injuries, level II and III hospitals can monitor and evaluate their provided care by comparison with the reference standard, visualized with the use of funnel and comet plots.

#### Compatibility with other trauma registries/uniform trauma registries world wide

Despite efforts at the Utstein consensus meeting, comparison of trauma registries remains restricted mainly due to the fact that there is significant variability of in- and exclusion criteria, resulting in selection bias or because not all trauma centers are obliged or willing to participate in the registry which results in non-nationwide representation [4, 15, 20]. A progressive next step would be to ensure that all trauma registers would be eligible for comparison, at least to some extent, which would allow countries to compare and learn from each other. Moreover, it might allow registries to be combined or collated to study European or even worldwide trauma care.

#### Conclusion

Participation and inclusion criteria differ greatly between European trauma registers, troubling international comparison or collaboration studies. This position paper is the first step toward an international guideline on how to use and interpret data using different international trauma databases starting with the DNTR.

## Supplementary Information

Below is the link to the electronic supplementary material.

Supplementary Table 1. Predictive model variables. Abbreviations: GCS: Glasgow Coma Scale; ED: Emergency Department; AIS: abbreviated injury score; ISS: Injury Severity Score (DOCX 30 KB)

Supplementary Table 2. System characteristic descriptors. Abbreviations: ED: Emergency Department; MMT: Mobile Medical Team (DOCX 20 KB)

Supplementary Table 3. Process mapping variables. Abbreviation: ED: Emergency Department; ICU: Intensive Care Unit (DOCX 22 KB)
