# Supplementary material for: Concepts, utilization, and perspectives on the Dutch Nationwide Trauma registry: a position paper
Source: Eur J Trauma Emerg Surg. 2023 Jan 9;49(4):1619–26. doi: 10.1007/s00068-022-02206-4 (PMC10449938; doi:10.1007/s00068-022-02206-4)
Supplement: Supplementary file 1 — Supplementary Table 1. Predictive model variables. Abbreviations: GCS: Glasgow Coma Scale; ED: Emergency Department; AIS: abbreviated injury score; ISS: Injury Severity Score (DOCX 30 KB) [file 68_2022_2206_MOESM1_ESM.docx]

| **Supplementary Table 1.** Predictive model variables | | | | |
| --- | --- | --- | --- | --- |
| Variable name | Type of data | Definition of data variable | Data variable categories or values | Percentage of missing values in 2021 (n=72361) |
| GESLACHTMAN | Nominal | Gender | 0 = Male  1 = Female  888 = Unknown  999 = Unknown, unchecked | 0.0% |
| LEEFTIJDSEHC | Continuous | The patients age at the time of injury (continuous) |  | 0.0% |
| LEEFTIJDSEH | Nominal | The patients age at the time of injury (in whole years) | 888 = Unknown  999 = Unknown, unchecked | 0.0% |
| LEEFTIJDSEHCAT | Nominal | The patients age at the time of injury (decades) | 0 = 0 - 9  1 = 10 – 19  2 = 20 – 29  3 = 30 – 39  4 = 40 – 49  5 = 50 – 59  6 = 60 – 69  7 = 70 – 79  8 = 80 – 89  9 = 90+ | 0.0% |
| COMORB | Ordinal | The comorbidity existing before the incident. Derangement resulting from the injury is not considered | 1 = a normal healthy patient  2 = a patient with mild systemic disease  3= a patient with severe systemic disease  4 = a patient with severe systemic disease that is a constant treat to life  5 = a moribund patient who is not expected to survive more than 24hours without an operation  888 = Unknown  999 = Unknown, unchecked | 12.0% |
| HARTSTILSTAND | Nominal | Prehospital cardiac arrest | 0 = No  1 = Yes  888 = Unknown  999 = Unknown, unchecked | 77.3% |
| REANIMATIE | Nominal | Prehospital cardiopulmonary resuscitation | 0 = No  1 = Yes  888 = Unknown  999 = Unknown, unchecked | 17.3% |
| OORZAAK | Nominal | Intention of injury | 1 = Accidental (unintentional)  2 = Self-inflicted (suspected suicide, incomplete suicide attempt, or injury attempt)  3 = Assault (suspected)  4 = Other  888 = Unknown  999 = Unknown, unchecked | 6.4% |
| LETSELWAARDEID |  | Dominating type of injury | 0 = Blunt  1 = Penetrating  888 = Unknown  999 = Unknown, unchecked | 6.4% |
| INTENTIE | Nominal | Mechanism of injury | 1 = Traffic accident: motor vehicle  2 = Traffic accident: motor bike  3 = Traffic accident: scooter  4 = Traffic accident: bicycle  5 = Traffic accident: pedestrian  6 = Traffic accident: other  7 = Gunshot injury  8 = Knifing injury  9 = Hitting with blunt object  10 = Low energetic trauma: low level falls  11 = High energetic trauma: fall from height  12 = Explosion  13 = Thermic injury  14 = Drowning  15 = Asphyxia  18 = Other  888 = Unknown  999 = Unknown, unchecked | 8.2% |
| OORZAAKCATEGORIEID | Nominal | Mechanism of injury (categorical) | 2 = Inflicted by others (violence)  4 = Traffic  5 = Work related injury  6 = Home  8 = Sport  9 = Self-harm  11 = Other  888 = Unknown  999 = Unknown, unchecked | 6.4% |
| TYPESPORT | String | Free text variable to define the type of activity that led to the injury |  | 0.0% |
| EYEOPENINGWAARDEID | Nominal | GCS eye movement component upon arrival in the ED/hospital | 1 = No eye response  2 = Opens eyes to pain  3 = Opens eyes to verbal command  4 = Opens eyes spontaneously  888 = Unknown  999 = Unknown, unchecked | 16.0% |
| MOTORRESPONSEWAARDEID | Nominal | GCS motor component upon arrival in the ED/hospital | 1 = No motor response  2 = Extension to pain  3 = Flexion to pain  4 = Withdraw from pain  5 = Localises pain  6 = Obeys commends/appropriate response to pain | 16.0% |
| VERBALRESPONSEWAARDEID | Nominal | GCS verbal component upon arrival in the ED/hospital | 1 = No verbal response  2 = Incomprehensible sounds  3 = Inappropriate words  4 = Confused  5 = Oriented | 16.0% |
| EMVCODETOTAAL | Nominal | First recorded pre-interventional GCS upon arrival |  | 16.2% |
| EMVCODE |  | First recorded pre-interventional GCS upon arrival in the ED/hospital (categorical) | 1 = 3  2 = 4 – 5  3 = 6 – 8  4 = 9 – 12  5 = 13 – 15 | 16.2% |
| ADEMFREQUENTIE | Continuous | Respiratory rate upon arrival in the ED/hospital | 888 = Unknown  999 = Unknown, unchecked | 26.2% |
| ADEMFREQUENTIEKLASSEID | Nominal | Respiratory rate upon arrival in the ED/hospital (categorical) | 0 = 0  1 = 1 – 5  2 = 6 – 9  3 = >29  4 = 10 – 29  888 = Unknown  999 = Unknown, unchecked | 26.2% |
| POLSFREQUENTIE | continuous | Heart rate upon arrival in the ED/hospital | 888 = Unknown  999 = Unknown, unchecked | 81.1% |
| SATURATIE | Nominal | Blood oxygen saturation upon arrival in the ED/hospital | 1 = 0  2 = 1 – 49  3 = 50 – 75  4 = 75 – 89  5 = >89 | 86.3% |
| RRDIASTOLISCH | Continuous | First recorded DBP upon arrival in the ED/hospital | 888 = Unknown  999 = Unknown, unchecked | 92.6% |
| RRSYSTOLISCH | Continuous | First recorded SBP upon arrival in the ED/hospital | 888 = Unknown  999 = Unknown, unchecked | 17.1% |
| SBPCODE | Nominal | First recorded SBP upon arrival in the ED/hospital (categorical) | 1 = 0  2 = 1 – 49  3 = 50 – 75  4 = 75 – 89  5 = >89 | 17.1% |
| INR | Nominal | International normalized ratio for measuring coagulation upon arrival in the ED/hospital | 777,7 = Not applicable  888 = Unknown  999 = Unknown, unchecked | 86.1% |
| INRR | Nominal | International normalized ratio for measuring coagulation arrival in the ED/hospital (categorical) | 0 = Abnormal value <0.8 or >1.2  1 = Normal value between 0.8 and 1.2 | 86.0% |
| BASE | Continuous | Arterial base excess upon arrival in the ED/hospital | 777,7 = Not applicable  888,8 = Unknown  999,8 = Unknown, unchecked | 92.8% |
| BASER | Nominal | Base excess (categorical) | 0 = No normal value  1 = Normal value (i.e., between -2.5 and 2.5) | 92.8% |
| BASEATLS | Nominal | Base excess (categorical) | 0 = >0  1 = Between 0 and -2  2 = Between -2 and -6  3 = Between -6 and -10  4 = Between -10 and -15  5 = ≤-15 | 96.3% |
| GEBRUIKALCOHOL | Nominal | Substance abuse (alcohol) measured upon arrival in the ED/hospital | 0 = No  1 = Yes  888 = Unknown  999 = Unknown, unchecked | 77.7% |
| PROMILAGE |  | If intoxicated with alcohol, permillage |  | 99.6% |
| GEBRUIKDRUG | Nominal | Substance abuse (drugs) measured upon arrival in the ED/hospital | 0 = No  1 = Yes  888 = Unknown  999 = Unknown, unchecked | 78.3% |
| HET | Nominal | High energetic trauma | 0 = No  1 = Yes  888 = Unknown  999 = Unknown, unchecked | 94.1% |
| RTSSEH | Nominal | Revised trauma score upon arrival in the ED/hospital |  | 38.6% |
| RTSSEHR | Nominal | Revised trauma score upon arrival in the ED/hospital (categorical) | 0 = 0  1 = 1  2 = 2  3 = 3  4 = 4  5 = 5  6 = 6  7 = 7  8 = 8  9 = 9  10 = 10  11 = 11  12 = 12 | 38.6% |
| RTSAMB | Nominal | Revised trauma score upon arrival of the ambulance |  | 84.2% |
| RTSAMBCAT |  | Revised trauma score upon arrival of the ambulance (categorical) | 0 = 0  1 = 1  2 = 2  3 = 3  4 = 4  5 = 5  6 = 6  7 = 7  8 = 8  9 = 9  10 = 10  11 = 11  12 = 12 | 38.6% |
| AISHEADGE3 | Nominal | The number of diagnosis in region of the head with a AIS between 3 and 6 |  | 0.9% |
| AISHEADGE4 | Nominal | The number of diagnosis in region of the head with a AIS between 4 and 6 |  | 0.9% |
| AISFACEGE4 | Nominal | The number of diagnosis in region of the face with a AIS between 4 and 6 |  | 0.9% |
| AISNECKGE4 | Nominal | The number of diagnosis in region of the neck with a AIS between 4 and 6 |  | 0.9% |
| AISSPINEGE4 | Nominal | The number of diagnosis in region of the spine with a AIS between 4 and 6 |  | 0.9% |
| AISUPPERGE4 | Nominal | The number of diagnosis in region of the upper extremities with a AIS between 4 and 6 |  | 0.9% |
| AISLOWERGE4 | Nominal | The number of diagnosis in region of the lower extremities with a AIS between 4 and 6 |  | 0.9% |
| AISTHORAXGE4 | Nominal | The number of diagnosis in region of the thorax with a AIS between 3 and 6 |  | 0.9% |
| AISEXTERNGE4 | Nominal | The number of diagnosis in external body region with a AIS between 3 and 6 |  | 0.9% |
| MAISHEAD | Nominal | Maximal AIS score in the region of the head |  | 0.9% |
| MAISFACE | Nominal | Maximal AIS score in the region of the face |  | 0.9% |
| MAISNECK | Nominal | Maximal AIS score in the region of the neck |  | 0.9% |
| MAISTHORAX | Nominal | Maximal AIS score in the region of the thorax |  | 0.9% |
| MAISABDOMEN | Nominal | Maximal AIS score in the region of the abdomen |  | 0.9% |
| MAISSPINE | Nominal | Maximal AIS score in the region of the spine |  | 0.9% |
| MAISUPPEREXTR | Nominal | Maximal AIS score in the region of the upper extremities |  | 0.9% |
| MAISLOWEREXTR | Nominal | Maximal AIS score in the region of the lower extremities |  | 0.9% |
| MAISEXTERN | Nominal | Maximal AIS score in the external region |  | 0.9% |
| SEVEREBURNS | Nominal | The patient has severe burns defined with an AIS score between 4 and 6 | 0 = No  1 = Yes | 0.9% |
| ISS | Nominal | Injury Severity Score |  | 1.0% |
| NISS | Nominal | New Injury Severity Score |  | 1.0% |
| ISSCAT | Nominal | Injury Severity Score category | 1 = 1 – 3  2 = 4 – 8  3 = 9 – 15  4 = 16 -24  5 = 25 – 49  6 = 50 – 66  7 = 75 | 1.0% |
| ISSCAT16 | Nominal | Polytrauma patient | 0 = No  1 = Yes  888 = Unknown  999 = Unknown, unchecked | 1.0% |
| AISNUM | Nominal | Number of AIS codes |  | 0.0% |
| AISNUMR | Nominal | Number of AIS codes (categorical) | 0 = 0  1 = 1  2 = 2  3 = 3  4 = ≥4 | 0.0% |
| NUMFRACT | Nominal | Number of fractures |  | 0.9% |
| HEUPFRAC | Nominal | Does the patient have a hip fracture and what is the injury severity score? | 0 = No  1 = Yes, and ISS 1-15  2 = Yes, and ISS >15 | 0.9% |
| GOSONTSLAG | Nominal | Glasgow Outcome Score at hospital discharge | 1 = Deceased  2 = Vegetive state  3 = Severe disability  4 = Moderate disability  5 = Good recovery  888 = Unknown  999 = Unknown, unchecked | 13.4% |
| OVERLEDEN | Nominal | Did the patient die during hospital admission? | 0 = No  1 = Yes  888 = Unknown  999 = Unknown, unchecked | 0.0% |
| MORTALITEIT | Nominal | Injury related mortality | 0 = Not deceased  1 = Deceased within 0 – 30 days after injury  2 = Deceased after more than 30 days | 18.0% |
| OVERLEDENMETOBDUCTIE | Nominal | Was there an autopsy performed | 0 = No  1 = Yes  888 = Unknown  999 = Unknown, unchecked | 76.4% |
| MORTALITEITDGN | Nominal | Time in days between arrival at the emergency Department and death (if deceased within 3 days) |  | 94.1% |
| DATUMOVERLEDEN | Date | Date and time of death | dd-mm-yyyy 00:00:00  01-01-1800 00:00:00 = Unknown, unchecked  08-08-1808 00:00:00 = Not available  09-09-1909 00:00:00 = Unknown | 94.2% |
| CHECKLEVEN | Date | Date last check survival status | dd-mm-yyyy 00:00:00  01-01-1800 00:00:00 = Unknown, unchecked  08-08-1808 00:00:00 = Not available  09-09-1909 00:00:00 = Unknown | 19.6% |
| OVERLEDEN30D | Nominal | Deceased between day 1 and day 30 after arrival at the ED/hospital | 0 = Not deceased  1 = Deceased within 1-30 days after injury  2 = Deceased >30 days after injury  888 = Unknown  999 = Unknown, unchecked | 18.0% |
| Abbreviations: GCS: Glasgow Coma Scale; ED: Emergency Department; AIS: abbreviated injury score; ISS: Injury Severity Score | | | | |
