# Supplementary material for: Concepts, utilization, and perspectives on the Dutch Nationwide Trauma registry: a position paper
Source: Eur J Trauma Emerg Surg. 2023 Jan 9;49(4):1619–26. doi: 10.1007/s00068-022-02206-4 (PMC10449938; doi:10.1007/s00068-022-02206-4)
Supplement: Supplementary file 2 — Supplementary Table 2. System characteristic descriptors. Abbreviations: ED: Emergency Department; MMT: Mobile Medical Team (DOCX 20 KB) [file 68_2022_2206_MOESM2_ESM.docx]

| **Supplementary Table 2.** System characteristic descriptors | | | | |
| --- | --- | --- | --- | --- |
| Variable name | Type of data | Definition of data variable | Data variable categories or values | Percentage of missing values in 2021 (n=72361) |
| DATUMOT | Date | Time of reporting at ambulance call centre | 00:00:00 | 100% |
| DATUMMKT | Date | Date and time of reporting at ambulance call centre | dd-mm-yyyy 00:00:00 | 53.0% |
| DATUMVT | Date | Date and time of ambulance dispatch | dd-mm-yyyy 00:00:00 | 53.6% |
| DATUMAPT | Date | Date and time of ambulance arrival at the scene | dd-mm-yyyy 00:00:00 | 53.15 |
| DATUMVPT | Date | Date and time of ambulance leaving the scene | dd-mm-yyyy 00:00:00 | 54.4% |
| DATUMAKTAMB | Date | Date and time of ambulance arrival at the hospital | dd-mm-yyyy 00:00:00 | 88.3% |
| REANIMATIE | Nominal | Pre-hospital cardiopulmonary resuscitation | 0 = no  1 = yes  888 = unknown  999 = unknown, unchecked | 17.3% |
| INTUBATIEPREHOSP |  | Pre-hospital intubation | 0 = no  1 = yes  888 = unknown  999 = unknown, unchecked | 19.3% |
| LUCHTWEG |  | Type of pre-hospital intubation | 0 = no pre-hospital intubation  1 = tube and medication  2 = tube without medication | 87.9% |
| VERKEERWAARDEID | Nominal | Ways of transportation from the site of injury to the hospital | 1 = ambulance  2 = patient came with own transportation  3 = helicopter emergency medical service  4 = ambulance with on scene support by air mobile medical team  5 = ambulance with on scene support by ground mobile medical team  6 = other  888 = unknown  999 = unknown, unchecked | 4.7% |
| INZETMMT | Nominal | Was a mobile medical team (MMT) dispatched | 0 = no  1 = yes  888 = unknown  999 = unknown, unchecked | 3.4% |
| MMT | Nominal | Type of mobile medical team (MMT) dispatched | 4 = helicopter  5 = ground-MMT  888 = unknown  999 = unknown, unchecked | 97.6% |
| TRAUMATEAMSEH | Nominal | Was the patient met by an activation of trauma team prior to or upon arrival at the hospital | 0 = no  1 = yes  2 = centre does not have a trauma team  888 = unknown  999 = unknown, unchecked | 8.6% |
| INTERVENTIETYPE | Nominal | Type of first key emergency intervention | 0 = no emergency intervention  1 = damage control thoracotomy  2 = damage control laparotomy  3 = extraperitoneal pelvic packing  4 = extremity revascularization  5 = intervention radiology  6 = craniotomy  7 = intercranial pressure monitoring  8 = coniotomy / cricothyrotomy  9 = other  10 = damage control orthopaedics  888 = unknown  999 = unknown, unchecked | 8.8% |
| INTERVENTIEDT | Date | Date of first key emergency intervention | dd-mm-yyyy 00:00:00 | 1.9% |
| ICJANEE | Nominal | Patient was admitted to the Intensive Care Unit | 0 = no  1 = yes | 0.6% |
| BEADEMINGR | Nominal | Days of mechanical airway support (categorical) | 1 = 1 day  2 = 2 days  3 = 3 – 7 days  4 = 8 – 14 days  5 = 15+ days  888 = unknown | 87.9% |
| LEVELHOOGHOSP | Nominal | Highest level of in-hospital care | 1 = Emergency Department  2 = general ward  3 = Operating theatre  4 = high Dependency Unit  5= Intensive Care Unit  888 = Unknown  999 = Unknown, unchecked | 3.0% |
| LEVELHOOGPREHOSP | Nominal | Highest level of pre-hospital care | 1 = No pre-hospital care provided  2 = Advanced Life support, no doctor on scene  3 = Advanced Life support, doctor on scene | 4.7% |
| VERVOERINTERKL | Nominal | Inter-hospital transfer | 0 = No  1 = Yes, transfer from another hospital  2 = Yes, transfer to another hospital  3 = Yes, transfer from and to another hospital  888 = Unknown  999 = Unknown, unchecked | 6.2% |
| OKJN | nominal | Was a surgical intervention performed? | 0 = No  1 = Yes  888 = Unknown  999 = Unknown, unchecked | 92.8% |
| OKAANTAL | Nominal | Number of surgical interventions performed | 888 = Unknown  999 = Unknown, unchecked | 17.1% |
| OVERPLBESTID | Nominal | In-hospital destination after | 1 = General ward  2 = Intensive care / high care / medium care  3 = Operating theatre  4 = Other hospital  5 = Deceased at the Emergency Department | 1.5% |
| SPECIALI | Nominal | Which department was in charge after ED resuscitation and admission to the ward | 1 = Trauma surgery  2 = Surgery  3 = Orthopaedics  4 = Neurosurgery  5 = Neurology  6 = Plastic surgery  7 = Cardio-thoracic surgery  8 = Cardiology  9 = Nose ear and throat  10 = Ophthalmology  11 = Paediatrics  12 = Paediatric surgery  13 = Internal medicine  14 = Oral and maxillofacial surgery  15 = Urology  16 = Psychiatry  17 = Pulmonology  18 = Pulmonary surgery  19 = Vascular surgery  20 = Geriatrics  21 = Gynaecology  22 = Other  23 = Burns  24 = Emergency department  888 = Unknown  999 = Unknown, unchecked | 72.4% |
| Abbreviations: ED: Emergency Department; MMT: Mobile Medical Team; | | | | |
