# Supplementary material for: Concepts, utilization, and perspectives on the Dutch Nationwide Trauma registry: a position paper
Source: Eur J Trauma Emerg Surg. 2023 Jan 9;49(4):1619–26. doi: 10.1007/s00068-022-02206-4 (PMC10449938; doi:10.1007/s00068-022-02206-4)
Supplement: Supplementary file 3 — Supplementary Table 3. Process mapping variables. Abbreviation: ED: Emergency Department; ICU: Intensive Care Unit (DOCX 22 KB) [file 68_2022_2206_MOESM3_ESM.docx]

| **Supplementary Table 3.** process mapping variables | | | | |
| --- | --- | --- | --- | --- |
| Variable name | Type of data | Definition of data variable | Data variable categories or values | Percentage of missing values in 2021 (n=72361) |
| ID | Number | Hospital specific identification number of admission |  | 0.0% |
| IDAABA | Number | Date of hospital admission |  | 0.0% |
| BA_IDX | Number | Region of injury |  | 0.0% |
| AA_IDX | Nominal | Geographical trauma region of injury | 80 = Network Acute care Limburg  116 = Network Acute Care Brabant  122 = Network Acute Care north-west  124 = Acute Care Euregio  126 = Acute Care Network North Netherlands  128 = Trauma centre West  130 = Trauma Network East Netherlands  132 = Trauma centre South-West Netherlands  134 = Network Acute Care Zwolle  479 = Spoedzorgnet | 0.0% |
| ONGEVALDT | Number | Date and time of injury |  | 0.0% |
| GPSDATA | String | Postal code of the scene of injury |  | 0.0% |
| HERKOMSTWAARDEID | Nominal | The patients last location before transportation to the ED/hospital | 1 = Scene of injury  2 = Other hospital  3 = Other care facility  4 = Foreign hospital  5 = General practitioners office  6 = Not applicable  888 = Unknown  999 = Unknown, unchecked | 2.2% |
| VERWYZER | Nominal | By who was the patient referred before arrival at the ED/hospital | 1 = 112 (national emergency number)  2 = General practitioner  3 = Self-referral  4 = Other hospital  7 = Out patient department  8 = Other  888 = Unknown  999 = Unknown, unchecked | 7.0% |
| JAARONGEVAL | Nominal | Year of injury | yyyy | 0.0% |
| MAANDONGEVAL | Nominal | Month of Emergency Department arrival | 1 = January  2 = February  3 = March  4 = April  5 = May  6 = June  7 = July  8 = August  9 = September  10 = October  11 = November  12 = December | 0.0% |
| UURONG | Nominal | Hour of injury |  | 20.0% |
| UURONGCAT | Nominal | Hour of injury (categorical) | 0 = morning (08:00 – 12:00)  1 = afternoon (12:00 – 17:00)  2 = evening (17:00 – 00:00)  3 = night (00:00 – 08:00) | 20.0% |
| AANRIJDTIJD | Continuous | Time required for Emergency Medical Services to get to the scene after emergency call | hh:mm:ss | 59.3% |
| BEHANDELTIJD | Continuous | Time spent on the scene by Emergency Medical Services | hh:mm:ss | 57.8% |
| VERVOERTIJD | Continuous | Time from departing scene until arrival at the ED/hospital | hh:mm:ss | 57.3% |
| TOTAALTIJD | Continuous | Total time between for Emergency call and arrival at the ED/hospital | hh:mm:ss | 64.6% |
| TOTAALTIJDONGEVAL | Continuous | Total time between injury and arrival at the ED/hospital (categorical) |  | 5.3% |
| TRAUMACENTRUM | Nominal | Patient was initially admitted to a level-I trauma centre | 0 = No, level-II or III trauma centre  1 = Yes, Level-I trauma centre | 0.0% |
| DAGAANKOMSTSEH | Nominal |  | 0 = Sunday  1 = Monday  2 = Tuesday  3 = Wednesday  4 = Thursday  5 = Friday  6 = Saturday  9 = Unknown | 0.0% |
| UURAANKSEH | Nominal | Hour of arrival at the Emergency Department | 99 = Unknown | 0.0% |
| UURAANKSEHCAT | Nominal | Hour of arrival at the Emergency Department (categorical) | 0 = Morning (08:00 – 12:00)  1 = Afternoon (12:00 – 17:00)  2 = Evening (17:00 – 00:00)  3 = Night (00:00 – 08:00) | 0.0% |
| VERBLIJFSDUURSEH | Nominal | Time spent in de Emergency Department |  | 6.5% |
| VERBLIJFSDUURSEHR | Nominal | Time spent in de Emergency Department (categorical) | 0 = 1 – 30 minutes  1 = 31 – 60 minutes  2 = 1 – 2 hours  3 = 2 – 3 hours  4 = 3 – 4 hours  5 = 4 – 14 hours | 6.8% |
| CTSCAN |  | Was a CT scan performed | 0 = No  1 = Yes  888 = Unknown  999 = Unknown, unchecked | 9.3% |
| CTSCANDT |  | Date and time of first CT scan |  | 61.0% |
| BEVERLOOPTIJD | Nominal | Time needed for base excess to be restored |  | 95% |
| INRR | Nominal | INR (categorical) | 0 = No normal value  1 = Normal value (i.e., between 0.8 and 1.2) | 86.1% |
| CTSCANTIJD | Continuous | Time from arrival at the Emergency Department and CT scan |  | 62.9% |
| UURVERTSEH | Nominal | Time when patient left the Emergency Room |  | 6.2% |
| UURVERTSEHCAT | Nominal | Time when patient left the Emergency Room (categorical) | 0 = Morning (08:00 – 12:00)  1 = Afternoon (12:00 – 17:00)  2 = Evening (17:00 – 00:00)  3 = Night (00:00 – 08:00) | 6.2% |
| DAGENOPNAME | Nominal | Days of admission |  | 3.2% |
| OPNAMEDUUR | Nominal | Days of admission (categorical) | 1 = 1 day  2 = 2 days  3 = 3 – 7 days  4 = 8 – 14 days  5 = 15 – 21 days  6 = >21 days | 3.2% |
| DAGENIC | Nominal | Number of days admitted to the intensive care | 888 = Unknown  999 = Unknown, unchecked | 0.8% |
| ICDUUR | Nominal | Days of Intensive Care admission (categorical) | 1 = 1 day  2 = 2 days  3 = 3 – 7 days  4 = 8 – 14 days  5 = 15+ days  888 = unknown | 0.8%% |
| BEADEMING | Nominal | Number of hospital admission days with mechanical ventilation | 777 = Not applicable, not admitted to the intensive care unit  888 = Unknown  999 = Unknown, unchecked | 87.9% |
| ONTSLAGBESTEMMINGID | Nominal | Destination after hospital discharge | 1 = Patients own home  2 = Retirement home / assisted living facility  3 = Nursing home  4 = Rehabilitation centre  5 = Other hospital  6 = Other care facility  7 = Foreign hospital  9 = Died in hospital  10 = Dismissed against medical advice  51 = Other hospital, higher level ICU  52 = Other hospital, same or lower-level ICU  53 = Other hospital, general ward  888 = Unknown  999 = Unknown, unchecked | 1.7% |
| Abbreviation: ED: Emergency Department; ICU: Intensive Care Unit; | | | | |
